# Selective neuronal expression of progranulin is sufficient to provide neuroprotective and anti-inflammatory effects after traumatic brain injury

**DOI:** 10.1186/s12974-024-03249-7

**Published:** 2024-10-10

**Authors:** Sudena Wang, Marc-Philipp Weyer, Regina Hummel, Annett Wilken-Schmitz, Irmgard Tegeder, Michael K. E. Schäfer

**Affiliations:** 1 Department of Anesthesiology, University Medical Center of the Johannes Gutenberg-University Mainz, Langenbeckstraße 1 (Bld. 505), 55131 Mainz, Germany; 2 Institute for Clinical Pharmacology, Faculty of Medicine, Goethe-University Frankfurt, Theodor Stern Kai 7 | Bd 74-75, Rm 4.101a, 60590 Frankfurt am Main, Germany; 3 Focus Program Translational Neurosciences (FTN) of the Johannes Gutenberg-University Mainz, Mainz, Germany; 4 Research Center for Immunotherapy (FZI) of the Johannes Gutenberg-University Mainz, Mainz, Germany

**Keywords:** Progranulin, Traumatic brain injury, Neuropathology, Neuroprotection, Neuroinflammation, Microglia, CD68, Therapy

## Abstract

Progranulin (PGRN), which is produced in neurons and microglia, is a neurotrophic and anti-inflammatory glycoprotein. Human loss-of-function mutations cause frontotemporal dementia, and PGRN knockout (KO) mice are a model for dementia. In addition, PGRN KO mice exhibit severe phenotypes in models of traumatic or ischemic central nervous system (CNS) disorders, including traumatic brain injury (TBI). It is unknown whether restoration of progranulin expression in neurons (and not in microglia) might be sufficient to prevent excessive TBI-evoked brain damage. To address this question, we generated mice with Nestin-Cre-driven murine PGRN expression in a PGRN KO line (PGRN-KO^*NestinGrn*^) to rescue PGRN in neurons. PGRN expression analysis in primary CNS cell cultures from naïve mice and in (non-) injured brain tissue from PGRN-KO^*NestinGrn*^ revealed expression of PGRN in neurons but not in microglia. After experimental TBI, examination of the structural brain damage at 5 days post-injury (dpi) showed that the TBI-induced loss of brain tissue and hippocampal neurons was exacerbated in PGRN-KO^*Grnflfl*^ mice (PGRN knockout with the mGrn fl-STOP-fl allele, Cre-negative), as expected, whereas the tissue damage in PGRN-KO^*NestinGrn*^ mice was similar to that in PGRN-WT mice. Analysis of CD68^+^ immunofluorescent microglia and *Cd68* mRNA expression showed that excessive microglial activation was rescued in PGRN-KO^*NestinGrn*^ mice, and the correlation of brain injury with *Cd68* expression suggested that *Cd68* was a surrogate marker for excessive brain injury caused by PGRN deficiency. The results show that restoring neuronal PGRN expression was sufficient to rescue the exacerbated neuropathology of TBI caused by PGRN deficiency, even in the absence of microglial PGRN. Hence, endogenous microglial PGRN expression was not essential for the neuroprotective or anti-inflammatory effects of PGRN after TBI in this study.

**Graphical Abstract:**

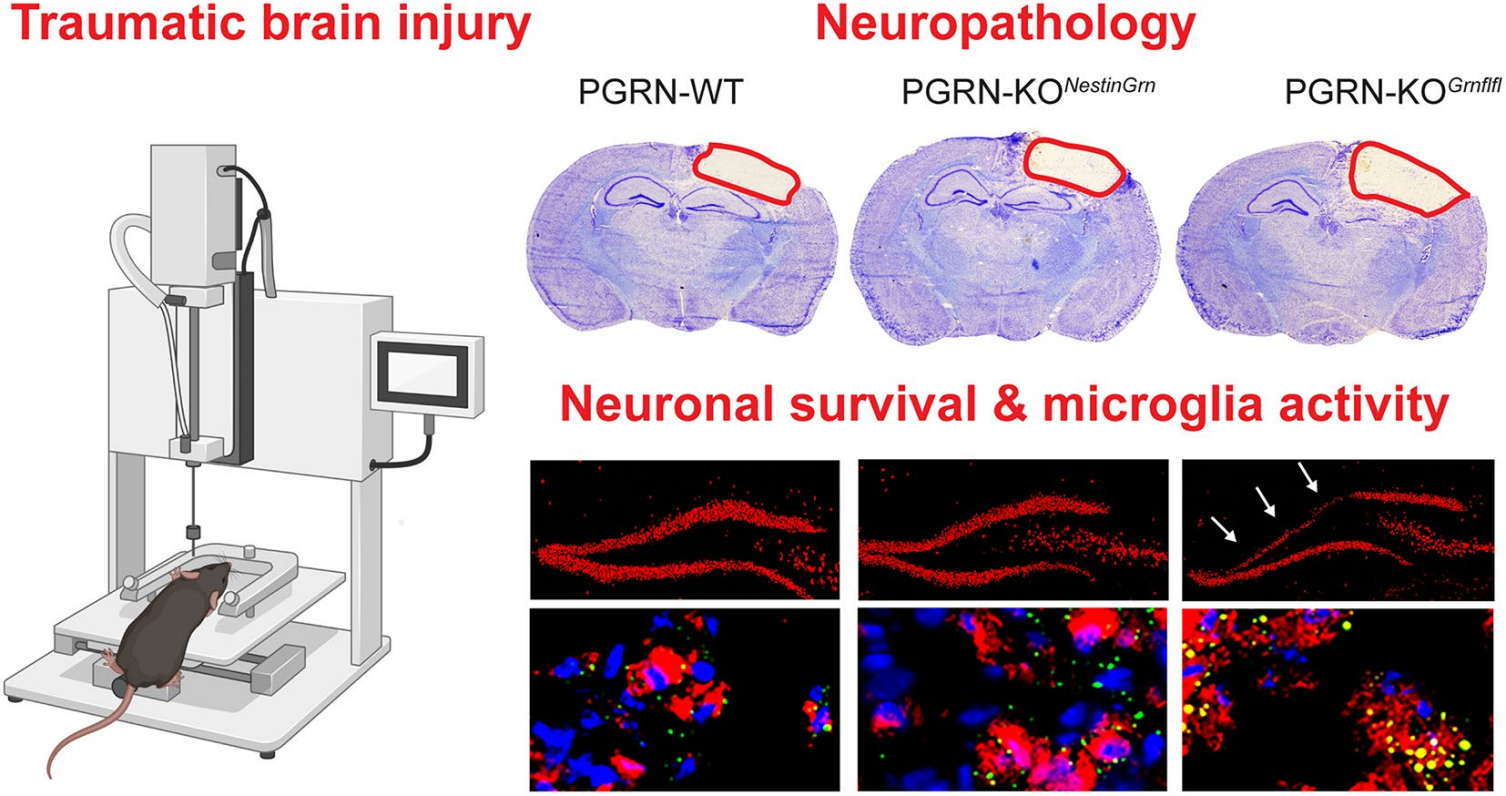

**Supplementary Information:**

The online version contains supplementary material available at 10.1186/s12974-024-03249-7.

## Background

Progranulin (PGRN) is a neurotrophic and anti-inflammatory factor expressed by neurons and microglia in the CNS [1]. Its functions are mediated by extracellular signaling via cell surface receptors such as Notch, EphA2, and SorCS2 [2–4]; inhibition of TNFα receptor signaling [5, 6]; and intracellular processes related to phagocytosis, autophagy, and lysosomal degradation [7, 8]. Extracellular PGRN was reported to be internalized and transported to lysosomes via the VPS10 domain-containing receptor sortilin [9], while other work has shown that lysosomal trafficking of PGRN occurs via intracellular routes [10].

Human loss-of-function mutations in the *GRN* gene are associated with neurodegenerative diseases, including frontotemporal dementia (FTD), neuronal ceroid lipofuscinosis, and Alzheimer’s disease [11–18]. Studies in *Grn*-deficient animal models suggested the therapeutic relevance of genetic or pharmacological approaches for restoring or enhancing PGRN expression [19–24]. These and other findings prompted clinical trials of gene therapy (NCT04747431, NCT04408625, and NCT06064890) or monoclonal antibody therapy (NCT04374136) to increase PGRN levels [20, 21]. However, no treatment effects of the PGRN elevating monoclonal antibody latozinemab were observed on the rate of clinical disease progression or on plasma biomarkers despite increasing PGRN levels 2-3-fold (NCT03987295) [25].

PGRN may also have therapeutic value in acute injury-induced neurodegeneration, such as traumatic brain injury (TBI), which is characterized by acute and delayed neuronal cell death and intense inflammatory responses by microglia, astrocytes and infiltrating peripheral immune cells [26–28]. Microglia are recognized as the primary mediators of the innate immune response, and their activation exacerbates and prolongs the secondary damage following TBI. However, substantial evidence also supports beneficial roles of microglia after the acute phase of TBI, contributing to clearance of cellular debris, resolution of central nervous system (CNS) inflammation and neural repair [29, 30]. Experimental studies from our laboratory and other laboratories demonstrated that TBI-induced brain damage is exacerbated in *Grn*-deficient mice [31–35]. A severe phenotype manifested as increased lysosomal biogenesis in microglia and increased numbers of degenerating neurons in *Grn*-deficient mice [34]. These findings are consistent with the anti-inflammatory and neuroprotective functions of PGRN in TBI. In experimental TBI rats, weighted gene coexpression analysis revealed [36] that upregulation of *Grn* expression was one of the prominent features of the microglial activation module [37]. Our own gene expression data from mice show that pharmacologic inhibition of TBI-induced proliferation of CD68^+^ microglia reduces PGRN mRNA expression [38] https://www.ncbi.nlm.nih.gov/geo/query/acc.cgi?acc=GSE196121. These findings indicate that the level of microglial PGRN outweighs that of neuronal PGRN after TBI.

However, the relative contribution of microglial or neuronal PGRN and whether enhancing PGRN functions is a therapeutic option in TBI are still unclear. We previously showed that intraventricular administration of recombinant PGRN shortly before injury induction attenuates the short-term consequences of TBI in PGRN-deficient mice [31] but not in wild-type mice [39], and a strong surplus of PGRN delivered by AAVs has been shown to result in deleterious effects in wild-type mice [40]. Conversely, significant improvements in neuronal PGRN overexpression were found in sciatic nerve injury models. Two- to fourfold PGRN overexpression in sensory neurons under the control of the Nav1.8 (SNS) promoter improved neuronal survival and recovery from sciatic nerve injury and pain in association with enhanced autophagy [4]. Improved peripheral nerve regeneration and reinnervation were also achieved in SLICK-Cre mice, in which PGRN was overexpressed by approximately 30% [41].

Here, we tested the hypothesis that transgenic Nestin-Cre-mediated expression of PGRN rescues exacerbated consequences in acute TBI in PGRN-deficient mice using the commonly used controlled cortical impact (CCI) model of TBI. CCI is a focal impact model that primarily mimics direct blunt trauma, producing pronounced cortical contusion followed by secondary processes of neurodegeneration and inflammation [42, 43]. Key readouts of neuropathology and inflammation were examined and compared at 5 days post-injury (dpi) between wild-type mice (PGRN-WT), mice with Nestin-Cre-driven PGRN expression on a PGRN-deficient background (PGRN-KO^*NestinGrn*^), and full PGRN knockout mice (PGRN-KO^*Grnflfl*^).

## Methods

### Animals

Animal studies were performed in compliance with the institutional guidelines of Johannes Gutenberg University, Mainz, Germany and were approved by the Animal Care and Ethics Committee of the Landesuntersuchungsamt Rheinland-Pfalz (protocol number 23177-07 G17-01-43). In this study, male adult 8- to 12-week-old PGRN-WT (*n* = 13), PGRN-KO^*Grnflfl*^ (*n* = 8), and PGRN-KO^*NestinGrn*^ (*n* = 8) mice were included. The age range of the mice corresponds approximately to young adult human, who are disproportionally often affected by TBI [44]. Only male mice were included to exclude the estrus-dependent hormonal status of female mice as possible confounding factors [45]. Mice were on a C57BL6/J genetic background as described [41] (*Nestin-Cre* (B6. Cg-*Tg (Nes-cre)1Kln*/J; Jackson Laboratory, #003771). Mice were housed individually and maintained in a controlled environment (12-hour dark/light cycle, 23 ± 1 °C, 55 ± 5% humidity) with food and water ad libitum. The researchers who performed the surgeries, data collection and analyses were blinded to the genotype identities.

PGRN-KO mice [46] (gifts from Aihao Ding, Grn^tm1Aidi^, MGI:4421704) were crossed with Gt(ROSA)26Sor^tm1.1(Ubc−Grn)Ite^ (MGI: 6149573), referred to as *Grnflfl* and with Nestin-Cre mice (B6. Cg-Tg (Nes-cre)^1Kln/J^). Triple heterozygous offspring were further crossed to homozygosity for the PGRN knockout allele and the mGrn fl-STOP-fl allele and to hemizygous Cre. Cre-positive mice exhibited neuronal PGRN expression, whereas Cre-negative animals had PGRN knockouts carrying an inactive floxed allele. A triple genotyping assay consisting of 6 probes was developed and is available from Transnetyx (strain: Nestin-GrnOE(-/-BG)). Details about the generation of the mouse line will be reported elsewhere.

## Preparation of microglia, neurons and astrocytes from the adult mouse brain

A Miltenyi Biotec Adult Brain Dissociation Kit and Miltenyi CD11b (Microglia) MicroBeads were used to isolate microglia. Briefly, mice were euthanized with CO_2_ and debleed via cardiac puncture, and the brains were collected in HBSS buffer. After washing with HBSS buffer, each brain was sliced into smaller pieces and collected in gentle MACS C tubes containing preheated enzyme mixture (buffer Z and enzyme P) at 37 °C. MACS enzyme mix (buffer Y and enzyme A) was added to each tube, which was subsequently incubated on a gentleMACS Octo dissociator at 37 °C and 50 rpm for 30 min. DNase I was subsequently added, followed by further incubation for 10 min at 37 °C and 50 rpm. HBSS buffer was added, and the tubes were incubated on ice for 5 min. Then, the samples were centrifuged, and the supernatant was removed. After resuspension in HBSS buffer, the samples were filtered through a 70 μm smart strainer, and the smart strainer was washed with HBSS buffer. The cell suspension was pelleted, and the supernatant was discharged. The pellet was resuspended in HBSS buffer and MACS debris removal solution, after which 4 ml of cold HBSS buffer was added, after which the mixture was subsequently centrifuged. The upper two phases were removed, followed by another centrifugation step. Finally, the pellet was resuspended in red blood cell removal solution and incubated at 4 °C for 10 min. PB buffer (0.5% BSA in HBSS) was added, the cells were again pelleted, and the supernatant was removed.

The cell pellet was then resuspended in PB buffer, 15 µl of MACS CD11b magnetic microbeads was added, mixed, and the mixture was incubated at 4 °C for 15 min. PB was added, after which the cells were pelleted. The supernatant was removed, and the pellet was resuspended in PB buffer.

MiniMACS MS columns were prepared with 500 µl of PB buffer and mounted on a magnetic stand (Miltenyi). The cell solution was added on top of the columns, and the columns were washed 3x with PB buffer. The flowthrough, containing non-microglial cells, mainly neurons and astrocytes, was collected and stored at -80 °C for later analysis. To elute microglia, 200 µl of PB buffer was added onto the columns and, after flow through, additional 800 µl PB buffer were pushed through the columns with the plunger. The collected cells were pelleted, and the supernatant was removed. The pellet was resuspended in 37 °C preheated medium (DMEM/F12-GlutaMax™ (200 µM) medium (Gibco) supplemented with 10% FCS and 1% PenStrep). The cells were counted in a Neubauer counting chamber, and approximately 50,000 cells were seeded into one well of an 8-well culture slide.

## Gene expression analysis using quantitative PCR

For *Grn* gene expression analysis in primary neural cells, RNA was isolated from the remaining microglia and neurons/astrocytes obtained during the isolation protocol. The Qiagen All Prep DNA/RNA/Protein Isolation Kit and the Qiagen RNA Isolation Kit were used according to the manufacturer’s instructions. The amount of isolated RNA and purity were measured on a Nanodrop spectrophotometer, and 180–200 ng of RNA and the Verso cDNA Synthesis Kit (Thermo Scientific) were used to generate cDNA via reverse transcription according to the manufacturer’s instructions. RT‒qPCR was conducted by using ORA™ SEE qPCR Green ROX (highQu) in duplicate on a QuantStudio™ 5 System (Thermo Fisher Scientific). Absolute values of Grn gene expression were normalized to the reference peptidylprolyl isomerase A (*Ppia*) and glyceraldehyde 3-phosphate dehydrogenase (*Gapdh*) values.

For gene expression analysis in brain tissues, samples were collected from cryosectioning (as described below), and gene expression was quantified as previously described [38]. Briefly, an RNeasy Kit (Qiagen) was used for mRNA extraction, and QuantiTect Reverse Transcription Kits (Qiagen) were used for cDNA synthesis. RT‒qPCR (Light Cycler 480, Hoffmann-La Roche AG RRID: SCR_012155) was performed in duplicate using Absolute Blue qPCR SYBR Green Mix (Thermo Fisher Scientific) or DyNAmo ColorFlash Probe qPCR. Absolute values of target gene expression were determined using a target-specific standard curve of mRNA copies and were normalized to the reference cyclophilin a (*Ppia*) expression.

Oligonucleotide sequences, amplicon sizes, annealing temperatures, and NCBI reference sequence numbers are provided in the Supplementary Material (Table S1).

## Experimental TBI

Experimental TBI using CCI was performed essentially as described [39]. Briefly, mice were subjected to general anesthesia with isoflurane (4 vol% induction, 2 vol% maintenance) and were immobilized in a stereotactic frame (Kopf Instruments). The rectal temperature was controlled at 37 °C by a feedback heating system (Hugo Sachs, MarchHugstetten, Germany). A 4 × 4 mm craniotomy was performed above the right parietal cortex, and the displaced bone fragment was flapped to one side while maintaining the integrity of the dura mater and avoiding bleeding. An electromagnetically driven CCI device (Benchmark™ Stereotaxic Impactor, Leica Biosystems, Wetzlar, Germany) was used to produce TBIs with an impactor tip diameter of 3 mm, a velocity of 6 m/s, a duration of 200 ms, and a displacement of 1.5 mm. Immediately following hemostasis, the displaced skull bone fragment was repositioned into the drill hole, histoacrylic glue was applied, and the wound was closed with filament sutures. After surgery, the mice were placed in a temperature controlled incubator (IC8000, Draeger, Luebeck, Germany) at 36 °C, allowed to awaken within 10 min and returned to the cages after approximately 1.5 h.

All animals survived the CCI procedure until the end of the observation period at 5 dpi. One animal from the WT group was excluded due to hydrocephalus observed during brain dissection after euthanasia. Two animals from the KO group were excluded from the study because of significantly abnormally high *Grn* mRNA expression, as detected by RT‒qPCR and verified using the ROUT outlier test. Body weight determined one day before TBI and 1, 3, and 5 dpi showed no differences between PGRN-KO^*Grnflfl*^ and PGRN-KO^*NestinGrn*^ mice, but a higher body weight of age- and background-matched C57BL/6J wild-type mice was observed in this study (Fig. S1).

## Brain tissue processing, histology, and immunofluorescence staining

Brain tissue processing was essentially performed as previously described [38]. Mice were decapitated under deep anesthesia using 4 vol% isoflurane at 5 dpi. The brains were carefully collected and frozen in powdered dry ice and kept at − 20 °C, and coronal sections of the brain were cut and collected using a cryotome (HM 560 Cryo-Star, Thermo Fisher Scientific, Walldorf, Germany). Brains were cut to 12-µm-thick slices across 16 consecutive levels at 500 μm intervals and collected on Superfrost Plus Adhesion Microscope slides (New Erie Scientific LLC, USA). Brain sections were taken from Bregma + 3.14 mm to − 4.36 mm. Intermediate sections (60 μm, 8 × 4) of brain tissue were taken from Bregma + 0.64 mm to − 2.86 mm. These sections were separated along the midline to obtain the right and left hemispheres. The upper quadrants of sections containing lesioned and perilesional brain tissue (cortex, striatum, dorsal hippocampus, and thalamus) were frozen in liquid nitrogen and subjected to RNA extraction. Cresyl violet staining was conducted to determine the brain volume and to assess structural brain damage. The brain sections were air-dried at room temperature (RT) for 1 h, rehydrated in 70% ethanol for 2 min and subsequently stained with a cresyl violet solution (10 mg/ml, 20% ethanol, Merck) for 10 min. The sections were rinsed in distilled water, dehydrated in 70%, 96% and 100% ethanol separately, hyalinized and subsequently mounted.

Immunofluorescence staining was performed with air-dried cryosections (RT for 30 min). Sections were fixed in 4% PFA for 10 min, washed in phosphate-buffered saline (PBS) and incubated with blocking solution (5% goat serum, 0.5% bovine serum albumin, 0.1% Triton X-100 in PBS) at RT for 1 h. Primary antibodies (Table S2) were applied to the blocking solution at 4 °C overnight. The next day, the sections were washed in PBS and incubated with secondary antibodies (Table S2) in blocking solution at RT for 1.5 h. BODIPY 493/503 (5 µg/ml, diluted from a 1 mg/ml stock solution in dimethylsulfoxide; Cayman) was used to identify neutral lipids in combination with antibody immunostaining and added after the completion of secondary antibody incubation and subsequent washing steps for 1 h at RT. After washing in PBS, the sections were counterstained with 4′,6-diamidino-2-phenyl-indol-dihydrochloride (DAPI, 1:10.000; Sigma‒Aldrich) and mounted in Immu-Mount (Fisher Scientific).

### Image acquisition and analysis

Images of sections stained with cresyl violet were captured using a bright field microscope (Stemi 305, Zeiss). Zen software was used for the quantification of lesion volume (Zeiss, RRID: SCR_013672) essentially as previously described [38]. Briefly, brain lesion volumes were determined by identifying areas without violet staining or absent tissue in the injured hemisphere from 16 consecutive brain cryosections and multiplying the intervals between two Sect. (500 μm). The relative lesion volume was calculated by dividing the lesion volume by the total ipsilateral hemisphere volume (Villapol et al., 2012).

Immunofluorescence images were captured using fluorescence microscopy (BZ-X800, Keyence) or confocal scanning microscopy (LSM Examiner, Zeiss) by researchers blinded to the genotype identities. ImageJ (ImageJ, RRID: SCR_003070) was used for quantitative analysis of cell counts and area with adequate threshold setting and the analyze particles plugin. Anti-NeuN^+^ cell counts in the hippocampal granule cell layer (GCL) were measured in the suprapyramidal blade of the dorsal hippocampus on three sections from Bregma levels − 1.86 mm to − 2.86 mm. The values are presented as percentages relative to the contralesional GCL.

### Statistical analysis

The data were analyzed with GraphPad Prism software (GraphPad Software, version 9.0). The data distribution was analyzed using the Shapiro‒Wilk normality test and QQ plots. Rout’s test was utilized to identify outliers, which were subsequently excluded from further evaluation, as specified in the figure legends. A comparative analysis of three groups was performed for parametric data by one-way analysis of variance (ANOVA) or Brown-Forsythe and Welch ANOVA tests depending on SD variance (F test); for nonparametric data, the Kruskal‒Wallis test was used. Holm-Šídák or Dunnett T3 or Dunn’s multiple comparison were used as post hoc tests for parametric or nonparametric data, respectively. Linear regression analyses were used to assess the association of ipsilesional mRNA expression values (RT‒qPCR) with % ipsilesional brain lesions or the GCL ratio (% ipsilesional or contralesional NeuN^+^ counts). Two-way ANOVA followed by the Holm–Šídák post hoc test was used for body weight comparisons. The values for individual animals are presented as the mean ± standard error of the mean (SEM). *p* < 0.05 was considered to indicate statistical significance; *p < 0.05, **p < 0.01, ***p < 0.001, ****p < 0.0001.

## Results

### Progranulin expression in PGRN-KO^*NestinGrn*^ mice

**Fig. 1:**
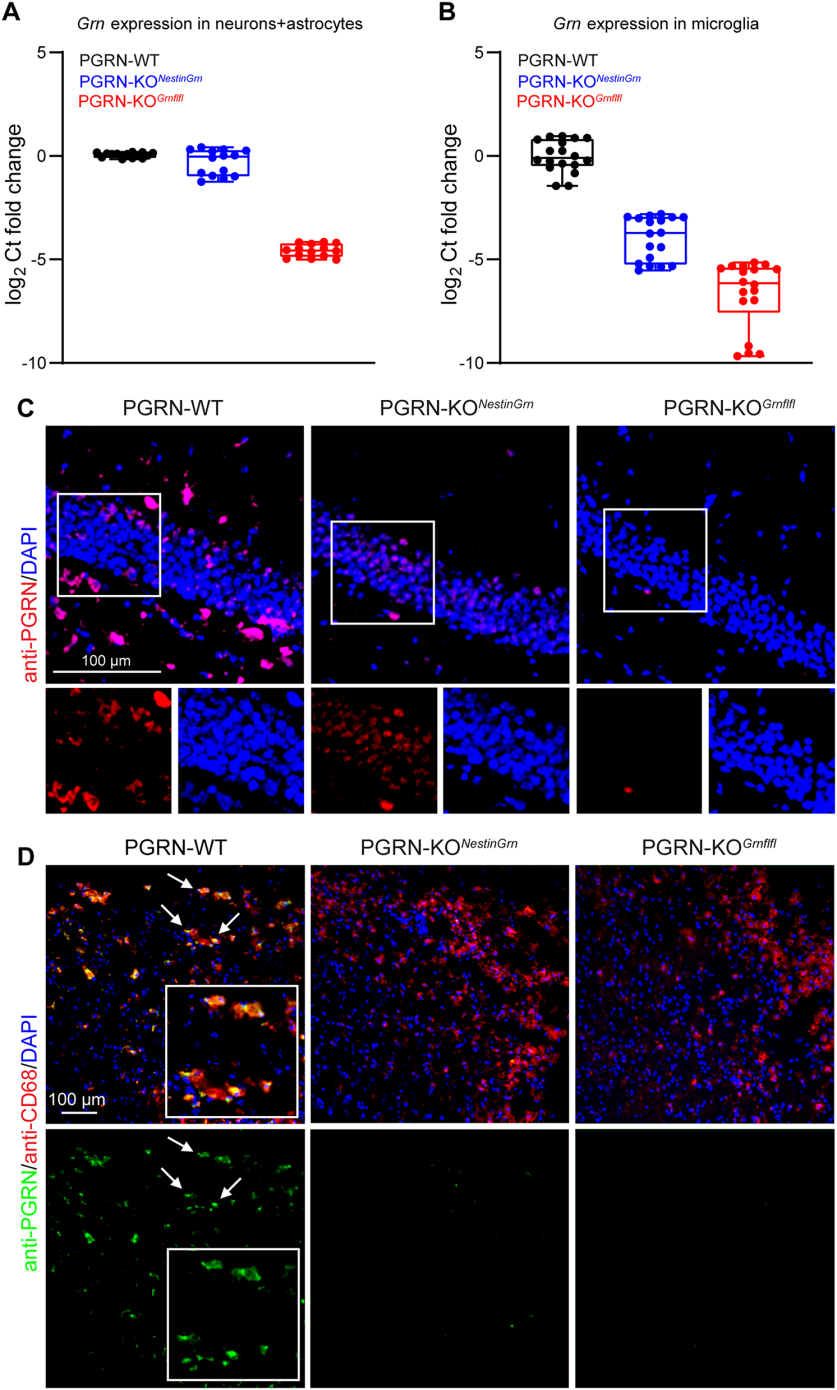
Progranulin expression in PGRN-KO^*NestinGrn*^ mice. (**A**, **B**) *Grn* mRNA expression in cell cultures from adult brains enriched for non-microglial neural cells (neurons and astrocytes) and microglia. The data are presented as box/scatter plots. The line is the median, the box shows the interquartile range, the whiskers show the minimum to maximum, and the scatters show the individual results of 7–9 mice per genotype, measured in duplicate. (**C**) Images of the contralesional hippocampal GCL (Bregma − 1.86 mm) showing anti-PGRN immunostaining and nuclear counterstaining by DAPI in PGRN-WT, PGRN-KO^*NestinGrn*^, and PGRN-KO^*Grnflfl*^ mice. Genotype-dependent PGRN expression in cells with microglial or neuronal morphology indicates PRGN expression in GCL neurons but not in microglia in PGRN-KO^*NestinGrn*^ mice. No specific signal was detected in PGRN-KO^*Grnflfl*^ mice. (**D**) Triple fluorescence staining of the ipsilesional cortex (Bregma − 1.86 mm) at 5 dpi using anti-PGRN/anti-CD68/DAPI revealed PGRN expression in CD68^+^ microglia in PGRN-WT mice but not in PGRN-KO^*NestinGrn*^ or PGRN-KO^*Grnflfl*^ mice. Arrows point to cells shown at higher magnification

Progranulin (PGRN) is a potent neurotrophic and anti-inflammatory factor constitutively expressed by mature neurons and activated myeloid cells such as microglia [1]. Upregulation of PGRN in CD68^+^ microglia was reported after trauma [35]; however, the relative contribution of microglial or neuronal PGRN to TBI is still unclear. To address this question, we generated mice with Nestin-Cre-driven PGRN expression in a PGRN KO line (PGRN-KO^*NestinGrn*^) to rescue PGRN in neurons but not in microglia. As expected, *Grn* mRNA expression analysis in primary CNS cell cultures (neurons, astrocytes, microglia) from PGRN-WT, PGRN-KO^*Grnflfl*^, and PGRN-KO^*NestinGrn*^ mice revealed that Nestin-Cre-driven Grn expression was predominant in neurons (Fig. 1A, B). Furthermore, we performed immunofluorescence staining of brain cryosections after experimental TBI at 5 dpi using an anti-PGRN antibody. In the non-injured, contralesional hippocampal dentate gyrus, immunostaining revealed PGRN^+^ microglia (based on morphology) in PGRN-WT mice and PGRN^+^ granule cells in PGRN-KO^*NestinGrn*^ mice (based on location), and absence of specific immunostaining in PGRN-KO^*Grnflfl*^ mice (Fig. 1C). To confirm PGRN expression in activated CD68^+^ microglia, we examined anti-PGRN immunostaining in the injured, ipsilesional cortex from PGRN-WT mice after experimental TBI at 5 dpi. PGRN expression was revealed in CD68^+^ activated microglia of PGRN-WT mice in brain lesions (lacking neurons) but not in those from PGRN-KO^*NestinGrn*^ or PGRN-KO^*Grnflfl*^ mice (Fig. 1D). Taken together, the newly generated PGRN-KO^*NestinGrn*^ mouse model expresses PGRN in neurons but lacks microglial PGRN expression.

### The exacerbated structural brain damage in PGRN-KO^*Grnflfl*^ mice is rescued in PGRN-KO^*NestinGrn*^ mice

**Fig. 2:**
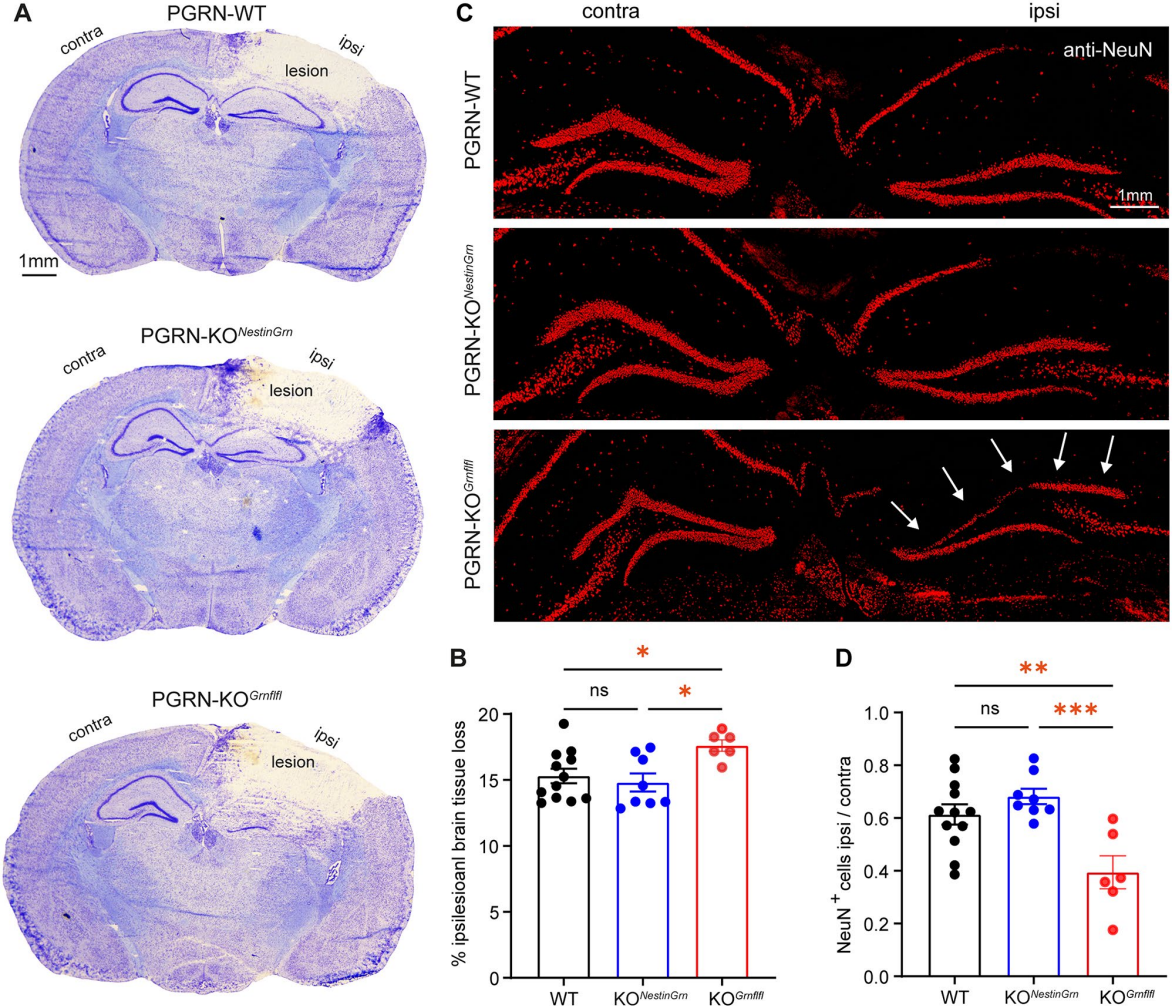
Exacerbated structural brain damage in PGRN-KO^*Grnflfl*^ mice is rescued in PGRN-KO^*NestinGrn*^ mice. (**A**) Representative images of cresyl violet-stained coronal brain sections from PGRN-WT, PGRN-KO^*NestinGrn*^, and PGRN-KO^*Grnflfl*^ mice at 5 dpi (Bregma − 1.86 mm). (**B**) Relative brain tissue loss (% of ipsilesional hemisphere) calculated from 16 consecutive sections (Bregma + 3.14 mm to − 4.36 mm). PGRN-WT and PGRN-KO^*NestinGrn*^ mice exhibit attenuated brain tissue loss compared to PGRN-KO^*Grnflfl*^ mice. (**C**) Images of coronal brain sections (Bregma − 1.86 mm) showing anti-NeuN immunostaining in the hippocampal dentate gyrus of the contra- and ipsilesional hemispheres at 5 dpi. Arrows indicate exacerbated loss of GCL neurons in the ipisilesional suprapyramidal blade of PGRN-KO^*Grnflfl*^ mice. (**D**) The number of NeuN^+^ neurons in the GCL was higher in PGRN-WT and PGRN-KO^*NestinGrn*^ mice than in PGRN-KO^*Grnflfl*^ mice. The data points represent individual mice, PGRN-WT (*n* = 12), PGRN-KO^*NestinGrn*^ (*n* = 8) and PGRN-KO^*Grnflfl*^ (*n* = 6), and the data are expressed as the mean ± SEM. One-way ANOVA with *Holm–Šidák* post hoc correction, *p < 0.05**p < 0.01, ***p < 0.001, ns = not significant

Previous studies demonstrated exaggerated inflammatory responses in PGRN-deficient mice after experimental TBI, but differences in structural brain damage were not consistently found [31, 33–35]. We therefore first determined the extent of the brain lesions at 5 dpi using cresyl violet-stained cryosections (Fig. 2A). Lesion volumetry across sixteen Bregma levels (+ 3.14 mm to − 4.36 mm) revealed genotype-dependent differences between PGRN-WT mice, PGRN-KO^*NestinGrn*^ and PGRN-KO^*Grnflfl*^ mice (Fig. 2B). PGRN-KO^*Grnflfl*^ mice had the largest brain lesions (17.61 ± 0.43, SEM, % of ipsilesional hemisphere), and the brain lesions were significantly smaller in both PGRN-WT mice (15.31 ± 0.54, SEM) and PGRN-KO^*NestinGrn*^ mice (14.81 ± 0.68, SEM), with no difference between these lines (Fig. 2B).

We next examined the dentate gyrus of the hippocampus, a brain region that is not directly affected by primary impact injury but shows secondary loss of neurons [47]. Anti-NeuN immunofluorescence staining was used to label neuronal cell bodies and nuclei and revealed neuronal loss in the ipsilesional suprapyramidal blade of the GCL, regardless of the genotype. However, neuronal loss was most pronounced in PGRN-KO^*Grnflfl*^ mice (Fig. 2C). To substantiate this observation, the numbers of NeuN^+^ cells in the ipsi- and contralesional suprapyramidal blades of the GCL were determined at three different Bregma levels, and ipsi- to contralesional ratios were calculated (Fig. 2D). The GCL ratio confirmed that neuronal loss (values < 1) was most pronounced in PGRN-KO^*Grnflfl*^ mice (0.39 ± 0.06, SEM) and was significantly attenuated in PGRN-WT mice (0.61 ± 0.03, SEM) and PGRN-KO^*NestinGrn*^ (0.68 ± 0.02, SEM) (Fig. 2D). Hence, the exacerbated structural brain damage in PGRN-KO^*Grnflfl*^ mice after experimental TBI was rescued in PGRN-KO^*NestinGrn*^ mice.

### Augmented TBI-induced gene expression of the inflammation-associated microglial marker *Cd68* in PGRN-KO^*Grnflfl*^ mice is partially rescued in PGRN-KO^*NestinGrn*^ mice

**Fig. 3:**
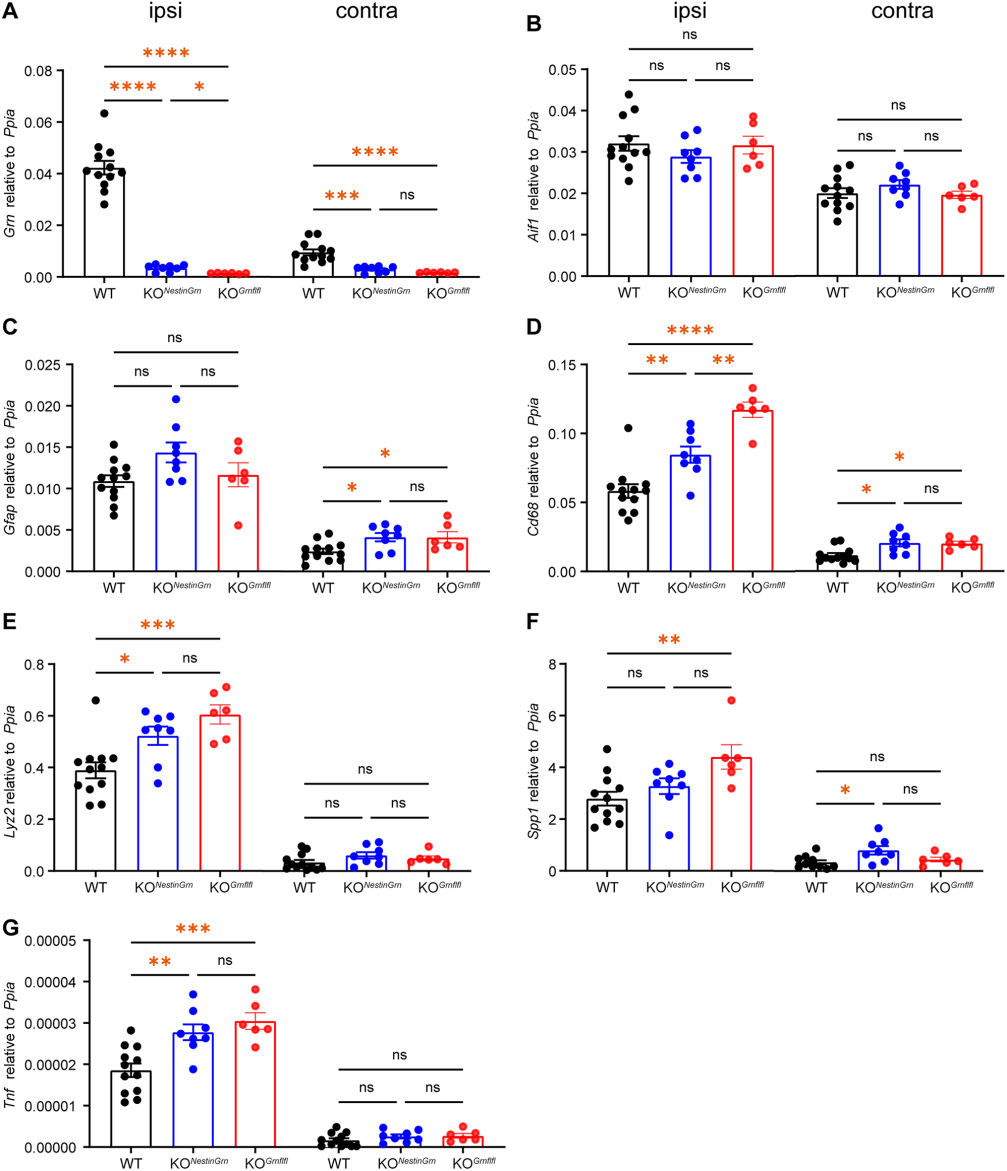
TBI-induced excessive *Cd68* gene expression in PGRN-KO^*Grnflfl*^ mice is attenuated in PGRN-KO^*NestinGrn*^ mice. (**A**-**G**) Gene expression analysis of inflammation-associated markers in ipsi- or contralesional brain tissues (Bregma + 0.64 mm to − 2.86 mm) was performed via RT‒qPCR. (**A**) *Grn* expression is highest in PGRN-WT mice and is reduced in PGRN-KO^*NestinGrn*^ and PGRN-KO^*Grnflfl*^ mice. *Grn* expression in ipsilesional brain tissue was mildly greater in PGRN-KO^*NestinGrn*^ mice than in PGRN-KO^*Grnflfl*^ mice but was substantially lower than that in WT mice because PGRN upregulation after TBI mainly occurs in microglia. (**B**-**G**) Column charts showing the mRNA expression of inflammation-associated markers (*Aif1*, *Gfap*, *Cd68*, *Lyz2*, *Spp1*, and *Tnf*). (**D**) Augmented ipsilesional *Cd68 *gene expression in PGRN-KO^*Grnflfl*^ mice was partially rescued in PGRN-KO^*NestinGrn*^ mice. Two outliers were identified by Rout’s test and excluded (**F**). The data points represent individual mice, PGRN-WT (*n* = 12), PGRN-KO^*NestinGrn*^ (*n* = 8) and PGRN-KO^*Grnflfl*^ (*n* = 6), and the data are expressed as the mean ± SEM. One-way ANOVA with *Holm–Šidák* post hoc correction, *p < 0.*05*, **p < 0.01, ***p < 0.001, **** *p* < 0.0001, ns = not significant

In addition to lesion volume, TBI outcome depends on the extent of inflammatory processes. Therefore, we examined the inflammatory response to TBI at 5 dpi. We quantified the relative gene expression levels of inflammatory markers, including *Grn*, via RT‒qPCR in samples from ipsi- and contralesional brain tissues (Fig. 3A‒G). While *Grn* expression was robustly induced by TBI in the ipsilesional brain tissue of PGRN-WT mice, it was almost undetectable in PGRN-KO^*Grnflfl*^*mice*, as expected, because PGRN is upregulated mainly in microglia, whereas its constitutive expression in neurons is not subject to adaptive upregulation at the transcriptional level. Consistent with the restrictive conditional genetic strategy, *Grn* expression was lower in PGRN-KO^*NestinGrn*^ mice than in PGRN-WT mice but higher than in PGRN-KO^*Grnflfl*^ mice (Fig. 3A, PGRN-WT: 0.042 ± 0.003, SEM; PGRN-KO^*NestinGrn*^: 0.003 ± 0.0005, SEM; PGRN-KO^*Grnflfl*^: 0.001 ± 0.00008, SEM). Similar results were obtained using ELISA to determine PGRN protein levels at 5 dpi, but the differences between PGRN-KO^*NestinGrn*^ and PGRN-KO^*Grnflfl*^ did not reach a statistically significant level (*p* = 0.1, PGRN-WT: 0.977 ± 0.055, SEM; PGRN-KO^*NestinGrn*^: 0.133 ± 0.007, SEM; PGRN-KO^*Grnflfl*^: 0.092 ± 0.014, SEM, Fig. S2).

Gene expression levels of the pan-microglia marker *Aif1* (encoding for Iba1) were increased in ipsilesional samples compared to contralesional samples, suggesting that PGRN deficiency did not affect the overall number of microglia after TBI (Fig. 3B). Similarly, ipsilesional gene expression of the reactive astrocyte marker GFAP did not differ between the genotypes, but contralesional samples from PGRN-KO^*NestinGrn*^ mice and PGRN-KO^*Grnflfl*^ mice showed increased *Gfap* expression compared to that of PGRN-WT mice (Fig. 3C).

We next determined the gene expression levels of the inflammation-associated microglial genes *Cd68*, *Lyz2*, *Spp1*, and *Tnf* (which encode CD68, Lysozyme 2, OPN, and TNFα, respectively). These genes were significantly upregulated in the ipsilesional samples of the PGRN-KO^*Grnflfl*^ mice compared to those of the PGRN-WT mice (Fig. 3D-G). Furthermore, the mean ipsilesional expression values were consistently lower in PGRN-KO^*NestinGrn*^ mice than in PGRN-KO^*Grnflfl*^ mice. However, statistical significance was only reached for the differential expression of *Cd68* (Fig. 3D), which exhibited a PGRN-KO^*Grnflfl*^ > > PGRN-KO^*NestinGrn*^ > PGRN-WT pattern, indicating that Nestin-Cre-driven PGRN expression was able to reduce but not fully abolish the exaggerated microglial activation caused by PGRN deficiency. The results from the contralesional samples were consistent with this conclusion, but it should be noted that our unilateral TBI model caused only mild inflammation-associated gene upregulation in the contralesional hemisphere.

### *Cd68* gene expression is a surrogate marker of microglial Grn deficiency and associated brain damage

We performed linear regression analyses to further study the relationship between the expression of inflammation-associated microglial genes (*Cd68*, *Lyz2*, *Spp1*, and *Tnf)* and brain tissue damage (brain tissue loss and the GCL ratio). Calculation of correlation coefficients and *p* values, independent of the genotype, revealed positive correlations between *Cd68*,* Lyz2*,* Spp1*, and *Tnf* and ipsilesional brain tissue loss and negative correlations with the GCL ratio. Gene expression showed stronger linear relationships with ipsilesional brain tissue loss than with the GCL ratio, as assessed by correlation coefficient (r^2^) and *p* value (Table S3).

**Fig. 4:**
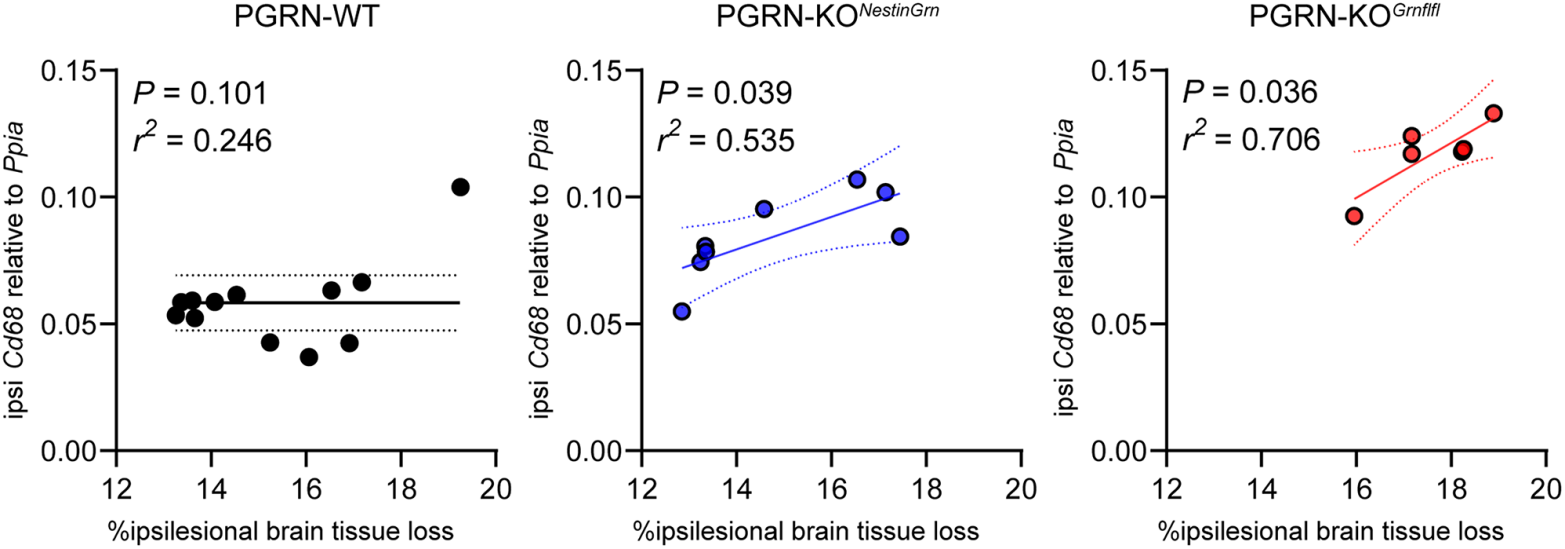
*Cd68* gene expression is a surrogate marker of microglial PGRN deficiency and associated brain damage. Linear regression analyses to assess the relationship between *Cd68* expression and ipsilesional brain tissue loss in PGRN-WT (*n* = 12), PGRN-KO^*NestinGrn*^ (*n* = 8), and PGRN-KO^*Grnflfl*^ (*n* = 6) mice. Scatter plots with regression lines, 95% confidence intervals, correlation coefficients (r^2^), and p values are shown. The data points represent individual mice

Strikingly, genotype-specific linear regression analyses revealed significant linear relationships between *Cd68* expression and ipsilesional brain tissue loss in PGRN-KO^*NestinGrn*^ and PGRN-KO^*Grnflfl*^ mice (p values < 0.05) and correlation coefficients (r^2^ values) in a PGRN-WT  ≺ PGRN-KO^*NestinGrn*^  ≺ PGRN-KO^*Grnflfl*^ pattern (Fig. 4 and Table S3). PGRN-KO^*NestinGrn*^ and PGRN-KO^*Grnflfl*^ mice lack PGRN expression in microglia. Therefore, these findings suggest that increased *Cd68* mRNA expression may serve as a surrogate marker of microglial *Grn* deficiency and associated brain damage.

### Excessive CD68^+^ microglial infiltration of the injured brain of PGRN-KO^*Grnflfl*^ mice is reduced in PGRN-KO^*NestinGrn*^ mice

**Fig. 5:**
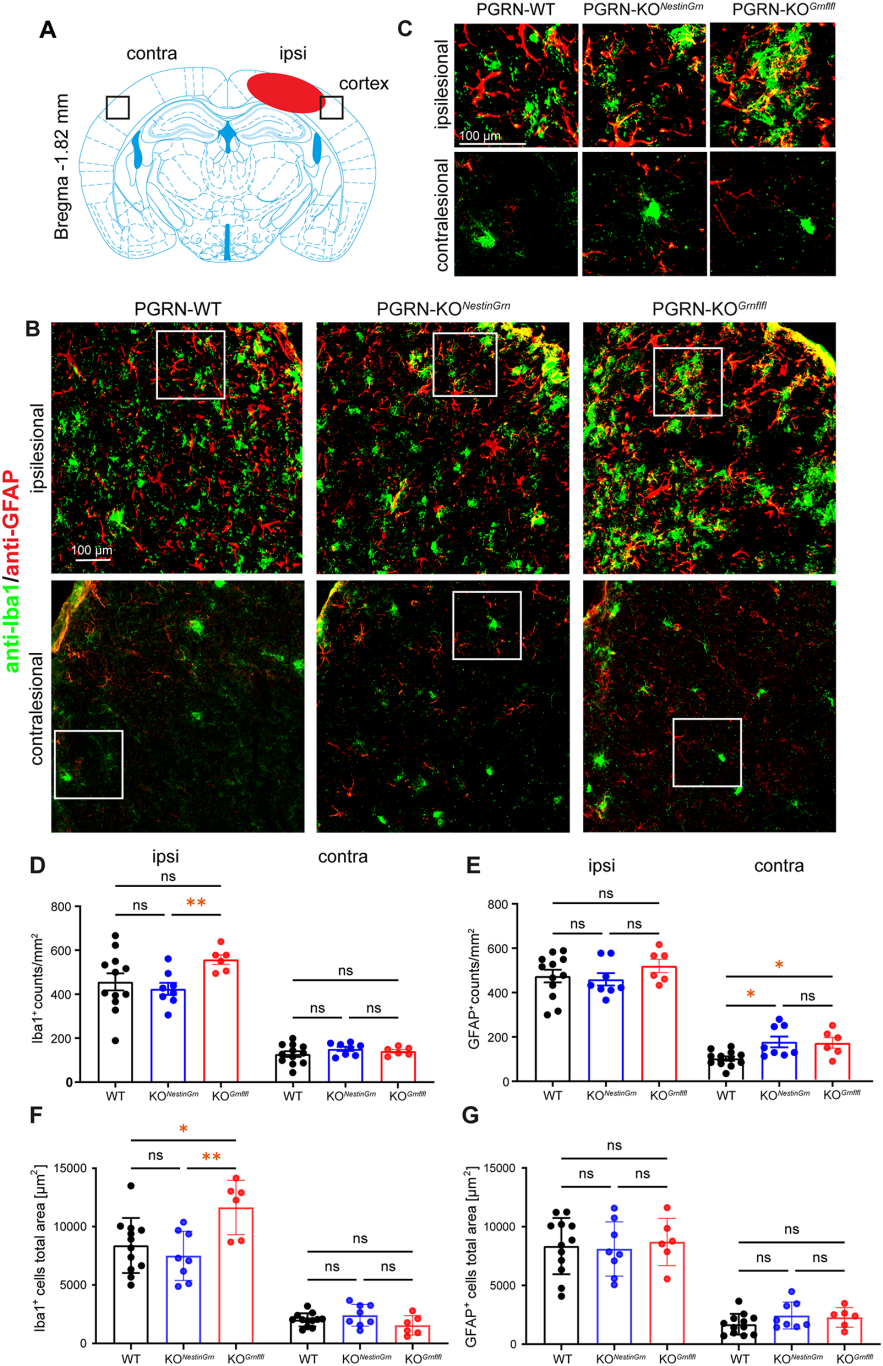
Iba1^+^ microglial infiltration of the injured brain in PGRN-KO^*Grnflfl*^ mice is reduced in PGRN-KO^*NestinGrn*^ mice. (**A**) Scheme illustrating the positions of the imaged brain regions. (**B**) Double immunostaining of the ipsi- and contralesional cortex at 5 dpi (Bregma − 1.86 mm) using anti-Iba1 and anti-GFAP antibodies showing TBI-evoked activation of microglia and astrocytes. (**C**) Higher magnification images of the boxed regions. (**D**, **E**) Column plots showing Iba1^+^ and GFAP^+^ counts in the ipsi- and contralesional cortices. The number of ipsilesional Iba1^+^ microglia was lower in PGRN-KO^*NestinGrn*^ mice than in PGRN-KO^*Grnflfl*^ mice, whereas the number of contralesional GFAP^+^ astrocytes was greater in PGRN-KO^*NestinGrn*^ and PGRN-KO^*Grnflfl*^ mice than in PGRN-WT mice. (**F**, **G**) Column plots showing the total anti-Iba1^+^ or anti- GFAP^+^ immunostained areas in the ipsi- and contralesional cortices. The area of ipsilesional Iba1^+^ immunostaining was smaller in PGRN-WT mice and PGRN-KO^*NestinGrn*^ mice than in PGRN-KO^*Grnflfl*^ mice, whereas the immunostaining area of GFAP^+^ astrocytes was not different between genotypes. The data are expressed as the mean ± SEM, and the values are shown for individual mice, PGRN-WT (*n* = 12), PGRN-KO^*NestinGrn*^ (*n* = 8) and PGRN-KO^*Grnflfl*^ (*n* = 6). One-way ANOVA (D, contra, E, ipsi), Brown-Forsythe ANOVA test (D, ipsi) and Kruskal‒Wallis test (E, contra) and post hoc *Holm–Šidák*, Dunnett T3 or Dunn’s corrections were used to calculate *p* values (*p < 0.05, **p < 0.01, ns = not significant)

**Fig. 6:**
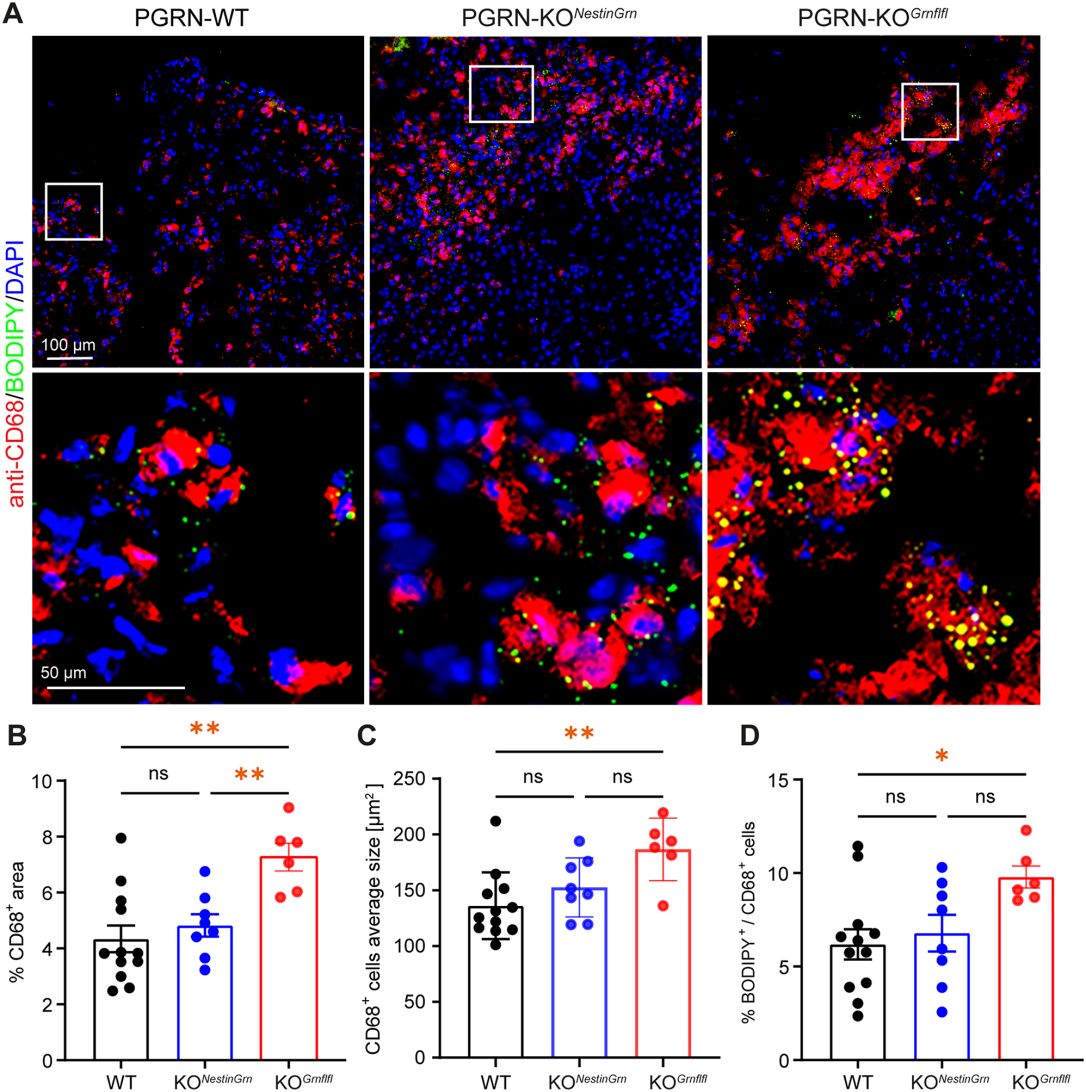
Excessive CD68^+^ microglial infiltration of the injured brain in PGRN-KO^*Grnflfl*^ mice is reduced in PGRN-KO^*NestinGrn*^ mice. (**A**) Triple-fluorescence staining of ipsilesional cortex at 5 dpi (Bregma − 1.86 mm) with anti-CD68/BODIPY/DAPI showed fewer CD68^+^ microglia in PGRN-WT and PGRN-KO^*NestinGrn*^ than in PGRN-KO^*Grnflfl*^ mice and partial overlap of the BODIPY signal with that in CD68^+^ microglia. (**B**) Column plots showing reduced area occupancy by CD68^+^ microglia in PGRN-WT and PGRN-KO^*NestinGrn*^ mice compared to PGRN-KO^*Grnflfl*^ mice. (**C**) Column plots showing reduced average size of CD68^+^ microglia in PGRN-WT mice compared to PGRN-KO^*Grnflfl*^ mice. Differences between PGRN-KO^*NestinGrn*^ mice and PGRN-KO^*Grnflfl*^ mice were statistically not significant (*p* = 0.07). (**D**) Column plots showing that the percentage of CD68^+^ microglia colabeled with BODIPY had the highest mean percentage in PGRN-KO^*Grnflfl*^ mice, a reduced mean percentage in PGRN-KO^*NestinGrn*^ and a significant reduction in PGRN-WT mice. The data are expressed as the mean ± SEM, and the values from individual mice are shown, PGRN-WT (*n* = 12), PGRN-KO^*NestinGrn*^ (*n* = 8) and PGRN-KO^*Grnflfl*^ (*n* = 6). One-way ANOVA and post hoc *Holm–Šidák* corrections were used to calculate *p* values (*p < 0.05, **p < 0.01, ns = not significant)

Microglia and astrocytes are strongly activated in response to TBI, which is even enhanced in the absence of PGRN, as observed in PGRN-deficient mice [31, 33, 35]. To further examine the impact of neuronal versus glial PGRN on this process, we performed double-immunofluorescence staining using specific antibodies against the pan-microglia marker Iba1 or the reactive astrocyte marker GFAP at 5 dpi and focused on the ipsi- and contralesional cortex (Fig. 5A). We observed a significant increase in Iba1^+^ microglia and GFAP^+^ astrocytes across all genotypes, which was greater in the ipsilesional cortex than at corresponding sites in the contralesional cortex, as expected (Fig. 5B, C). There were no statistically significant differences in the number of Iba1^+^ microglia between PGRN-WT and PGRN-KO^*Grnflfl*^ mice, but the number of ipsilesional Iba1^+^ microglia was lower in PGRN-KO^*NestinGrn*^ mice than in PGRN-KO^*Grnflfl*^ mice (Fig. 5D). In addition, the total anti-Iba1^+^ immunostained area in the ipsilesional cortex was significantly increased in PGRN-KO^*Grnflfl*^ compared to PGRN-WT and PGRN-KO^*NestinGrn*^ mice (Fig. 5F). Interestingly, compared with those in WT mice, more contralesional GFAP^+^ astrocytes were observed in the cortices of both PGRN-KO^*NestinGrn*^ and PGRN-KO^*Grnflfl*^ mice (Fig. 5E). However, the total anti-GFAP^+^ immunostained area was not different between the three genotypes (Fig. 5G). As detailed above, our data suggest that non-microglial PGRN expression can partially compensate for the loss of anti-inflammatory PGRN in CD68^+^ microglia. CD68 is a phagolysosomal compartment marker, and its expression by microglia together with lipid droplet (LD) formation after TBI define the proinflammatory and phagocytic active state of microglia [48, 49]. We examined this relationship depending on PGRN genotypes and combined anti-CD68 immunostaining with LD staining using the neutral lipid dye BODIPY. CD68^+^ microglia colabeled with BODIPY were observed at cortical lesion sites and in the surrounding perilesional tissue (Fig. 6A). We determined the area occupied by CD68^+^ immunostained cells and their average size in images of ipsilesional lesion sites in the cortex. This analysis revealed a significant increase in brain infiltration of CD68^+^ cells in PGRN-KO^*Grnflfl*^ mice compared to PGRN-KO^*NestinGrn*^ and PGRN-WT mice (Fig. 6B). In addition, the average size of CD68^+^ cells differed in a PGRN-WT  ≺ PGRN-KO^*NestinGrn*^ ≺ PGRN-KO^*Grnflfl*^ pattern (Fig. 6C) and the differences between PGRN-WT and PGRN-KO^*Grnflfl*^ were statistically significant. Next, we calculated the percentage of CD68^+^ cells with LD formation and found that PGRN-KO^*Grnflfl*^ mice had the highest number of double anti-CD68/BODIPY-positive microglia. The ratio of double-positive cells was significantly greater in these mice than in PGRN-WT mice (Fig. 6D), whereas the difference between PGRN-KO^*Grnflfl*^ mice and PGRN-KO^*NestinGrn*^ showed a statistical trend, but did not reach statistical significance (*p* = 0.078, one-way ANOVA, Fig. 6D). Consistent with the *Cd68* gene expression data, these results suggest that Nestin-Cre-mediated PGRN expression in non-microglial cells (but with deficiency of PGRN in microglia themselves) attenuates the appearance of proinflammatory and phagocytic CD68^+^ microglia after TBI and has a mild effect on LD formation in these cells.

## Discussion

We showed in the present study that Nestin promoter-driven PGRN expression in neurons on a PGRN knockout background (PGRN-KO^*NestinGrn*^ mice) partially rescues the excessive structural damage that occurs after experimental TBI in full PGRN knockout mice (PGRN-KO^*Grnflfl*^). In addition, brain tissue infiltration by CD68^+^ microglia was attenuated in PGRN-KO^*NestinGrn*^ mice, although the microglia themselves lacked PGRN. We also provided evidence that *Cd68 *is a surrogate marker of “PGRN deficiency-associated” brain tissue damage. The results of the present study suggest that endogenous PGRN expression in microglia is not essential for attenuating structural brain damage during the acute phase of experimental TBI and that relevant neuroprotective effects at this early post-traumatic period are achieved by restoring neuronal PGRN expression. Low neuronal expression was sufficient for this rescue. These findings may have potential implications for understanding the cell type-specific functions of PGRN and for therapeutic approaches for TBI and beyond.

In this study, the CCI model of TBI was used, which induces a highly reproducible pattern of injury that mimics a direct blunt trauma [42, 43]. We studied mice at 5 dpi because the primary cortical contusion injury progresses and excessive inflammatory activation of microglia occurs in the first week after CCI [50, 51]. Furthermore, we previously observed upregulation of CCI-induced Grn mRNA expression at 5 dpi (but not after 1 dpi), which was associated with increasing numbers of microglia [31]. In addition, Tanaka et al. using a stab wound lesion model and immunofluorescence staining reported similar expression regulation of PGRN, which colocalized with the microglial markers Iba1 and CD68 [35]. Therefore, the posttraumatic time point of 5 dpi using the CCI model was considered appropriate in the present study to test the hypothesis that transgenic Nestin-Cre-mediated expression of PGRN rescues exacerbated consequences in acute TBI in PGRN-deficient mice. However, it should be noted that no single animal model of TBI mimics all aspects of clinical TBI [52] and that focusing exclusively on the acute phase after experimental TBI does not provide conclusions about the subacute and long-lasting chronic phases.

Nevertheless, during the acute phase and contrary to our expectations, microglial-derived PGRN was not essential for the rescue of severe TBI-induced brain damage caused by PGRN deficiency, although microglia are the main source of PGRN in the CNS. Several studies have reported the upregulation of PGRN in microglia after brain insult in humans and in animal models [1, 53, 54], and single-nucleus sequencing revealed that PGRN deficiency promotes a disease-associated state of microglia correlated with neurodegeneration in mice [55]. As described here and elsewhere [35], compared with WT mice, PGRN-deficient mice exhibit increased infiltration of injured brain tissue by CD68^+^ microglia after TBI. However, the reduction in brain tissue infiltration by CD68^+^ microglia in PGRN-KO^*NestinGrn*^ mice compared to that in PGRN-KO^*Grnflfl*^ mice was remarkable, as neither of these mouse lines exhibited microglial PGRN expression. Hence, we conclude that Nestin-driven PGRN expression confers neuroprotection.

Several studies have reported the neuroprotective effects of neuronal PGRN expression in neuronal *Cre* transgenic mice [4, 22, 41]. It is conceivable that neurons that express PGRN, albeit at low levels, are less vulnerable to TBI-induced damage, resulting in reduced brain tissue loss and reduced damage-associated CD68^+^ microglial infiltration. The hypothesis that the neuropathological phenotype of PGRN-KO^*Grnflfl*^ mice depends on neuronal dysfunction in the absence of PGRN rather than exaggerated microglial activation is supported by previous studies in which aged PGRN-KO mice were used as a model of FTD/NCL [23, 56]. The authors demonstrated that adenoviral-mediated neuronal PGRN expression leads to a reduction in neuropathological lipofuscinosis and microgliosis [23], and depletion of microglial PGRN did not aggravate the FTD/NCL-like phenotypes of mice deficient in neuronal PGRN [56].

The rescue of PGRN deficiency in TBI and FTD animals likely shares similar mechanisms, which may apply to a cell-autonomous, neurocentric model, i.e., neuronal PGRN protects neurons. An alternative explanation, not mutually exclusive, would be a non-cell-autonomous, cooperative model. Soluble PGRN is actively released by intact neurons or passively released by injured neurons or other non-microglial cell types and might be taken up by microglia in PGRN-KO^*NestinGrn*^ mice to suppress proinflammatory activation. This model is consistent with previous observations in aged PGRN mice crossed with *LysM-cre* mice [56]. Also, our results showing a reduced microglial pro-inflammatory phenotype in PGRN-KO^*NestinGrn*^ mice compared to PGRN-KO^*Grnflfl*^ suggest that soluble PGRN exerts effects independent of its cellular origin. Overexpression of PGRN by microglia or other cell types might be also beneficial, if the extracellular PGRN would be taken up by neurons and microglia to fulfil functions equal to those of PGRN produced within the cell, which is however still not proven. Uptake of soluble PGRN by intact or injured neurons, would fit a non-cell-autonomous model.

The finding that sufficient effects were achieved even at low expression levels of neuronal PGRN raises questions regarding the dose-dependent effects of soluble PGRN and their impact on signaling of cell surface receptors known to interact with PGRN, such as TNF receptors [5], Notch [41], EphA2 [2], SorCS2 [3], and Sortilin [9]. These studies reported different binding affinities of PGRN to these receptors, suggesting that their activation may be affected differently by the availability of soluble PGRN. This may also be related to our observation that the number of astrocytes was increased in the non-injured, contralesional hemisphere in PGRN-KO^*NestinGrn*^ mice and PGRN-KO^*Grnflfl*^ lacking microglial PGRN as a major source of soluble PGRN. Likewise, overexpression of PGRN or administration of recombinant PGRN may also result in different receptor activation patterns after TBI. For example, EphA2 has been associated with exacerbated ischemic brain injury and blood-brain barrier damage [57, 58] and a compromised blood-brain barrier was observed following intracerebroventricular administration of high dose recombinant PGRN in the CCI model of TBI [39]. Future studies should investigate the dose-dependent effects of PGRN and the associated activation of receptors in more detail, and consider potential regional expression differences.

According to immunostaining-based techniques, the expression of PGRN is lower in neurons in the mouse brain than in microglia or other myeloid cells [1, 59, 60]. In situ hybridization studies have shown stronger *Grn* expression in neurons [61] (gensat https://www.gensat.org/bgem_ish.jsp?probe_id=2937). However, using a commercial polyclonal antibody directed against full-length recombinant PGRN expressed by HEK293 cells (Sino Biological, Cat: 50396-RP02), we did not observe consistent neuronal anti-PGRN immunostaining in PGRN-WT mice, including in the hippocampal GCL, but rather did observe anti-PGRN immunolabeling of GCL neurons in PGRN-KO^*NestinGrn*^ mice. The low abundance of neuronal PGRN and proteolytic processing of secreted PGRN [59] may contribute to the difficulty in detecting neuronal PGRN in the adult mouse brain via antibody-based immunofluorescence techniques.

There are potential limitations regarding the use of the Nestin-Cre mouse line in this study. Nestin-Cre mice are a well-studied model for switching on/off gene expression in neuronal cells during early development [62], first described in 1999 [63], with more than 1000 references in the database resource Mouse Genome Informatics for this line (MGI:2176173). Nestin is a marker of neuronal progenitor cells, and its expression starts early during embryogenesis around E10. We used Nestin-Cre to cut off a floxed STOP codon in front of mouse progranulin to switch on progranulin in Nestin-positive cells, which are mainly neuronal cells in the CNS gray matter. Physiologic progranulin expression also starts during embryogenesis presumably a few days after Nestin expression, and its expression in neurons increases during maturation [1]. Hence, the expression of our PGRN transgene was not completely synchronized with the physiologic neuronal expression of progranulin. In addition, Nestin-Cre mice were reported to have few health issues per se, such as decreased body weight [64]. Although we did not observe differences in body weight between PGRN-KO^*Grnflfl*^ (no *cre* transgene) and PGRN-KO^*NestinGrn*^ mice, the age- and background-matched C57BL/6J wild-type mice used in this study had a slight but significantly greater body weight, which is probably not explained by the genotype but might have caused an unexpected bias.

A major limitation of this study is the examination of a single post-traumatic time point at 5 dpi. Focusing exclusively on the acute phase after experimental traumatic brain injury precludes conclusions about the subacute and long-lasting chronic phases and provides only limited insights into the complex pathogenesis following TBI. Previous studies have demonstrated long-lasting microglial activation [65, 66] and distinct microglial subpopulations that adopted longitudinal changes in the expression of various molecular markers, likely reflecting dynamic changes in microglial function [67, 68]. Consequently, it will be important to test whether the early neuroprotective and anti-inflammatory effects of neuronal PGRN expression persist beyond the acute and subacute phases of TBI, as well as to conduct a comprehensive characterization of microglial subpopulations, including CD68^+^ microglia subtypes. Another limitation is that only male mice were investigated. Future studies should also include female mice, as sex-specific differences in central and peripheral immune cells have been reported resulting from PGRN-deficiency in aged mice [69]. Moreover, studies focusing on behaviour would be crucial as protective effects on brain tissue do not always correspond to functional outcomes [70, 71].

In conclusion, our results on PGRN expression confirm previous observations that microglia are the main source of PGRN in adult mouse brain tissue, especially after brain injury, and that neurons express low levels of PGRN [1, 7, 36]. Therefore, our approach using C57BL/6J wild-type, PGRN-KO^*Grnflfl*^ and PGRN-KO^*NestinGrn*^ mice was sufficient to draw conclusions on the contribution of microglial and neuronal PGRN to TBI pathogenesis. According to the aforementioned data obtained from aged PGRN-deficient mice [23, 56], we propose that neuronal PGRN was sufficient in the present study to provide neuroprotection after TBI. It remains speculative whether higher Nestin-Cre-mediated PGRN expression is associated with stronger neuroprotection or rather adverse effects of arbitrarily high neuronal PGRN or extensive Cre expression. Given a potential therapeutic value of PGRN in TBI and other CNS disorders, it will be essential to further investigate conditional mouse models suitable to control cell type-specific, dose-dependent and spatiotemporal effects of PGRN on the activation of different cell surface receptors, microglial activation and neuronal survival. In this context, the characterization of the cell-autonomous and non-cell-autonomous functions of PGRN is crucial.

## Electronic supplementary material

Below is the link to the electronic supplementary material.

Supplementary Material 1

## Data Availability

The datasets generated and analysed during the current study are included in this published article or available from the corresponding author on reasonable request.
