# Supplementary material for: Selective neuronal expression of progranulin is sufficient to provide neuroprotective and anti-inflammatory effects after traumatic brain injury
Source: J Neuroinflammation. 2024 Oct 10;21:257. doi: 10.1186/s12974-024-03249-7 (PMC11468377; doi:10.1186/s12974-024-03249-7)
Supplement: Supplementary file 1 — Supplementary Material 1 [file 12974_2024_3249_MOESM1_ESM.docx]

## Supplementary Material

## Table S1: Oligonucleotide primers

| Gene name, (amplicon size, annealing temperature) | Oligonucleotide sequences 5′–3′  (fw: forward, rev: reverse) | Gene bank number |
| --- | --- | --- |
|  | | |
| Brain tissue analysis | | |
| *Ppia* (146 bp, 58°C) | fw- GCGTCTSCTTCGAGCTGTT  rev- RAAGTCACCACCCTGGCA | NM_008907 |
| *Grn* (145 bp, 58°C) | fw- ATGCTGTGTGCTGTGAGGAC  rev- CACTCCACATTCCCAACCTT | NM_008175 |
| *Cd68* (113 bp, 58°C) | fw- CCCACCTGTCTCTCTCATTTC  rev- CACATTGTATTCCACCGCC | NM_001291058.1 |
| *Aif1* (144 bp, 58°C) | fw- ATCAACAAGCAATTCCTCGATGA  rev- CAGCATTCGCTTCAAGGACATA | NM_019467 |
| *Gfap* (120 bp, 58°C) | fw- CGGAGACGCATCACCTCTG  rev- TGGAGGAGTCATTCGAGACAA | NM_001131020 |
| *Spp1* (151 bp, 58°C) | fw- ATGTCATCCCTGTTGCCCAG  rev- GACTGATCGGCACTCTCCTG | NM_001204201.1 |
| *Lyz2* (237 bp, 58°C) | fw- ACTCCTCCTGCTTTCTGTC  rev- TTGCCATCATTACACCAGTATC | NM_017372.3 |
| *Tnfa* (212 bp, 62°C) | fw- TCTCATCAGTTCTATGGCCC  rev- GGGAGTAGACAAGGTACAAC | NM_013693 |
| Cell culture analysis | | |
| *Ppia* (144 bp, 60°C) | fw- GCTGGACCAAACACAAAACGG  rev- GCCATTCCTGGACCCAAAAC | NM_008907 |
| *Gapdh* (100 bp, 60°C) | fw- CCTCGTCCCGTAGACAAAATG  rev- TCTCCACTTTGCCACTGCAA | NM_001289726.2 |
| *Grn* (171 bp, 60°C) | fw- CTGCCCGTTCTCTAAGGGTG  rev- ATCCCCACGAACCATCAACC | NM_008175 |

## Table S2: Antibodies

| Primary antibodies | | | | | |
| --- | --- | --- | --- | --- | --- |
| Primary antibody | Host | IHC, dilution | | Manufacturer | RRID |
| CD68 | Rat | 1:750 | | Bio-Rad Laboratories, Inc.; Hercules; CA, USA | AB_324217 |
| Iba1 | Guinea pig | 1:500 | | Synaptic System GmbH; Göttingen, DE | AB_2924932 |
| NeuN | Guinea pig | 1:1,500 | | Synaptic System GmbH; Göttingen, DE | AB_2924930 |
| GFAP | Rabbit | 1:1,000 | | Dako North America, Inc.; Carpinteria, CA, USA | AB_10013382 |
| Progranulin | Rabbit | 1:1000 | | Sino Biological Europe GmbH; Eschborn, DE | No RRID available, Cat: 50396-RP02 |
| Secondary antibodies | | | | | |
| Fluorophore | Goat anti- | IHC, dilution | Manufacturer | | RRID |
| Alexa 488 | guinea pig | 1:500 | Invitrogen/Life Technologies | | AB_2534117 |
| Alexa 488 | Rabbit | 1:500 |  |  | AB_2576217 |
| Alexa 568 | rat | 1:500 |  |  | AB_2534121 |
| Alexa 568 | Guinea pig | 1:500 |  |  | AB_2534119 |
| Alexa 568 | Rabbit | 1:500 |  |  | AB_143157 |
| Alexa 633 | Rabbit | 1:500 |  |  | AB_2535731 |

## Table S3: Linear regression analyses


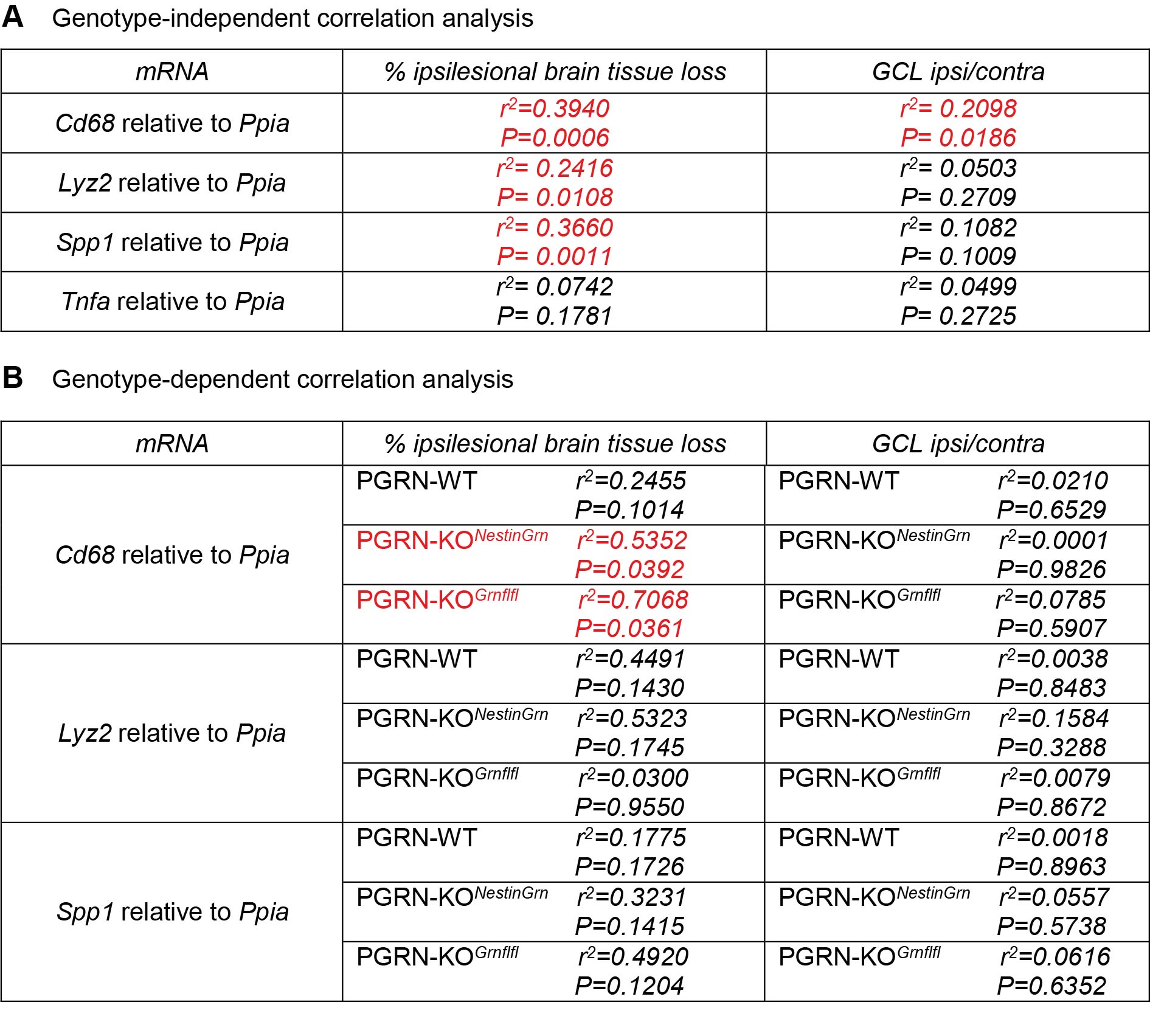


## Figure S1: Body weight


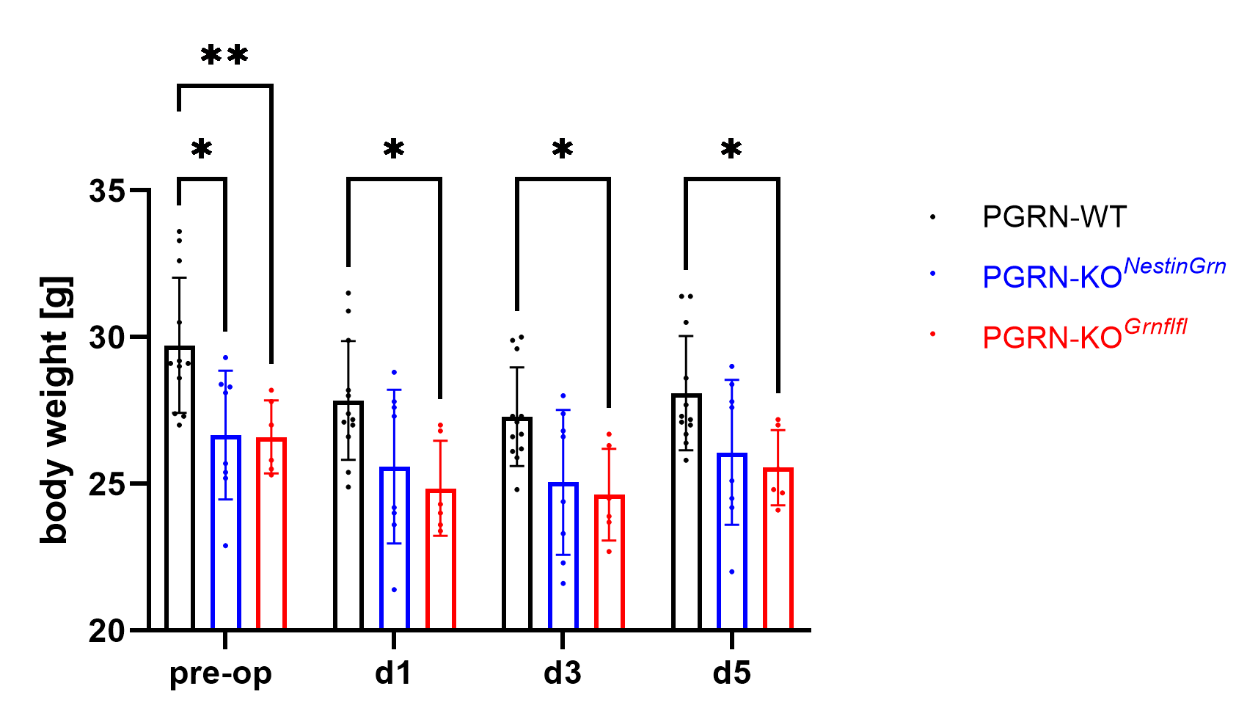


Figure S1: Body weight determined one day before TBI and 1, 3, and 5 dpi showed no differences between PGRN-KO*^Grnflfl^* and PGRN-KO*^NestinGrn^* mice and a slight but significantly higher body weight of PGRN-WT (age- and background-matched C57BL/6J wild-type) mice. Data are expressed as mean ± SEM and values from individual mice are shown. Two-way ANOVA and post-hoc Holm-Šidák corrections were used to calculate *p* values (**p*<0.05, ***p*<0.01).

## Figure S2: Enzyme Linked Immunosorbent Assay (ELISA)


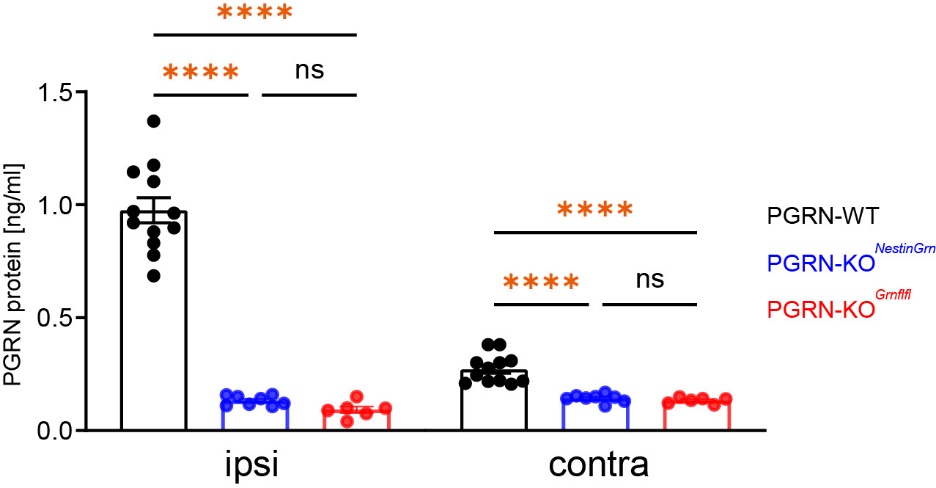


Figure S2: Column plots showing PGRN protein expression from ipsi- and contralesional brain tissue samples using anti-PGRN ELISA according to the manufacturer`s instructions (Adipogen, AG-45A-0019Y). PGRN protein levels at 5 dpi is highest in PGRN-WT mice and is reduced in PGRN-KO*^NestinGrn^* and PGRN-KO*^Grnflfl^* mice (PGRN-WT: 0.977 ± 0.055, SEM; PGRN-KO*^NestinGrn^*: 0.133 ± 0.007, SEM; PGRN-KO*^Grnflfl^*: 0.092 ± 0.014, SEM). PGRN protein expression in ipsilesional brain tissue was mildly greater in PGRN-KO*^NestinGrn^* mice than in PGRN-KO*^Grnflfl^* mice (*p*=0.1). The data points represent individual mice, PGRN-WT (n=12), PGRN-KO*^NestinGrn^* (n=8) and PGRN-KO*^Grnflfl^* (n=6), and the data are expressed as the mean ± SEM. Brown-Forsythe ANOVA test and post hoc Dunnett T3 were used to calculate *p* values, **** *p*<0.0001, ns=not significant.
